# Apolipoprotein M promotes cholesterol uptake and efflux from mouse macrophages

**DOI:** 10.1002/2211-5463.13157

**Published:** 2021-05-02

**Authors:** Shuang Yao, Fan Zheng, Yang Yu, Yuxia Zhan, Ning Xu, Guanghua Luo, Lu Zheng

**Affiliations:** ^1^ Clinical Medical Research Center the Third Affiliated Hospital of Soochow University Changzhou China; ^2^ Xiangyang Central Hospital Affiliated Hospital of Hubei University of Arts and Science Xiangyang China; ^3^ Section of Clinical Chemistry and Pharmacology Institute of Laboratory Medicine Lund University Sweden

**Keywords:** apolipoprotein M, atherosclerosis, cholesterol, high‐density lipoprotein, Scavenger receptor class B type I

## Abstract

Apolipoprotein M (ApoM) exhibits various anti‐atherosclerotic functions as a component of high‐density lipoprotein (HDL) particles. Scavenger receptor class B type I (SR‐BI) is a classic HDL receptor that mediates selective cholesterol uptake and enhances the efflux of cellular cholesterol to HDL. However, the effect of ApoM on cholesterol transport in macrophages remains unclear. In this study, we identified for the first time that ApoM is expressed in mouse macrophages and is involved in cholesterol uptake, similar to SR‐BI. NBD‐cholesterol uptake and efflux in cells were characterized using fluorescence spectrophotometry. The uptake ratios of cholesterol by macrophages from *ApoM* ^−/−^ *SR‐BI^−/−^* mice were significantly lower than those from *ApoM^+/+^SR‐BI* ^−/−^ and *ApoM* ^−/−^ *SR‐BI^+/+^* mice. Real‐time fluorescence quantitative PCR was used to analyze the expression of cholesterol transport‐related genes involved in cholesterol uptake. ApoM‐enriched HDL (ApoM^+^HDL) facilitated more cholesterol efflux from murine macrophage Ana‐1 cells than ApoM‐free HDL (ApoM^−^HDL). However, recombinant human ApoM protein inhibited the ability of ApoM^−^HDL to induce cholesterol efflux. In conclusion, ApoM promotes cholesterol uptake and efflux in mouse macrophages. A better understanding of ApoM function may lead to the development of novel therapeutic strategies for treating atherosclerotic diseases.

## Graphical abstract

In this study, we mainly explore the role of ApoM in two aspects: the uptake and efflux of cholesterol in mouse macrophages. ApoM deficiency leads to decreased cholesterol uptake by mouse peritoneal macrophages. ApoM^+^HDL promotes cellular cholesterol efflux more efficiently than ApoM^−^HDL does. It is quite possible that ApoM promotes cholesterol efflux by SR‐BI‐dependent mechanisms.

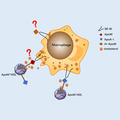

**Abbreviations**
ABCA1: ATP‐binding cassette transporter A1
ABCG1: ATP‐binding cassette transporter G1
ApoA‐I: Apolipoprotein A‐I
ApoE: Apolipoprotein E
ApoM: Apolipoprotein M
ApoM^+^HDL: ApoM‐enriched HDL
ApoM^‐^HDL: ApoM‐free HDL
DKO: *ApoM* ^‐/‐^ *SR‐BI* ^‐/‐^ double‐knockout
HDL: High‐density lipoprotein
RCT: Reverse cholesterol transport
Rh‐ApoM: Recombinant human ApoM protein
SR‐BI: Scavenger receptor class B type I
WT: Wild‐type

Apolipoprotein M (ApoM) was first discovered by Xu in 1999 [1]. Similar to apolipoprotein A‐I (ApoA‐I), ApoM is predominantly associated with high‐density lipoprotein (HDL). ApoM, which constitutes ~ 5% of plasma HDL particles [2], plays an important role in protecting endothelial barrier function [3] and affects several potential anti‐atherogenic pathways, such as reverse cholesterol transport (RCT) [4, 5], the formation of preβ‐HDL [6] and removal of reactive oxygen species [7]. A recent study showed that ApoM‐enriched HDL (ApoM^+^HDL) promotes cellular cholesterol efflux from porcine brain capillary endothelial cells more efficiently than ApoM‐free HDL (ApoM^−^HDL), while ApoM silencing diminished cellular cholesterol release [8]. ApoM^+^HDL contains significantly more free cholesterol than ApoM^−^HDL, suggesting that ApoM regulates cholesterol metabolism possibly by promoting increased lipid transport by HDL [2].

Scavenger receptor class B type I (SR‐BI), the first cell surface protein to be characterized as an HDL receptor [9], is abundant in the liver and macrophages. Several experiments have demonstrated that HDL induces the efflux of cholesterol from foam cells via SR‐BI [10, 11]. Macrophage SR‐BI is critical to atheroprotective effects because macrophages are the most abundant cell type in atherosclerotic lesions, and macrophage SR‐BI expression affects cholesterol homeostasis and inflammation [12]. Pagler *et al* demonstrated that SR‐BI is a receptor that facilitates the uptake of HDL particles. Internalization and subsequent resecretion of HDL are important for the efflux of cellular cholesterol [13]. Fuentes *et al*. showed that insulin promotes cholesterol uptake by SR‐BI‐dependent mechanisms in a model of the human intestinal epithelium [14]. However, the role of SR‐BI in mediating cholesterol efflux from mouse macrophages is controversial [15, 16]. Therefore, the purpose of the present study was to ascertain the roles of primary murine macrophage ApoM and SR‐BI in cholesterol uptake and efflux.

Macrophage accumulation within the subendothelium or neointima constitutes one of the first steps in atherogenesis. The key to this process is the scavenging of excess cholesterol by macrophages that take up and store cholesterol until it can be mobilized from these cells by components of the HDL system. Therefore, in our study, we chose Ana‐1 murine macrophage cells and four types of primary murine macrophages with different genotypes as research models. ApoM is a component of HDL, and SR‐BI is the HDL receptor. Deletion of either the *ApoM* or *SR‐BI* gene leads to a disturbance in cholesterol homeostasis. However, the impact of knockout of both *ApoM* and *SR‐BI* on cholesterol transport in murine macrophages is not clear. Therefore, we crossed the *ApoM* and *SR‐BI* single‐gene knockout mice for generations to generate *ApoM* ^−/−^ *SR‐BI* ^−/−^ double‐knockout mice (DKO). The cholesterol uptake capacity of macrophages from different genotypes was further compared.

ATP‐binding cassette transporter A1 (ABCA1) and ATP‐binding cassette transporter G1 (ABCG1), two key cholesterol transporters in macrophages, function in mediating cellular cholesterol efflux and contributing to RCT [17, 18]. ABCA1 facilitates intracellular phospholipids and cholesterol efflux to lipid‐free ApoA‐I [19], whereas ABCG1 mediates intracellular oxysterol and cholesterol efflux to HDL [20]. Kober *et al*. suggested that ABCG1, SR‐BI, and ApoM are centrally involved in cholesterol efflux from porcine brain capillary endothelial cells [8]. However, the relationship between the ATP‐binding cassette transporter and ApoM or SR‐BI in macrophages has not yet been elucidated.

The present study aimed to clarify the influence of *ApoM* and/or *SR‐BI* gene deletion on macrophage cholesterol uptake. Subsequently, we compared the macrophage cholesterol efflux capacity to identify whether there was any difference between ApoM^+^HDL and ApoM^−^HDL. This better understanding of ApoM function may yield novel therapeutic strategies for treating atherosclerotic diseases.

## Materials and methods

### Chemicals and reagents

FBS and RPMI 1640 medium were purchased from Gibco (Auckland, New Zealand). BSA (fatty acid‐free) was purchased from MP Biomedicals (Santa Ana, CA, USA). Phorbol‐12‐myristate‐13‐acetate (PMA) was obtained from Gene Operation (Ann Arbor, MI, USA). The fluorescent sterol 22‐(N‐(7‐nitrobenz‐2‐oxa‐1,3‐diazol‐4‐yl)‐amino)‐23,24‐bisnor‐5‐cholen‐3β‐ol (NBD‐cholesterol), NBD, and Triton X‐100 were purchased from Sigma (St. Louis, MO, USA). Specific and high‐affinity monoclonal antibody against human ApoM was prepared and identified by Abgent Biotech (Suzhou, China). In the process of synthesizing ApoM recombinant protein *in vitro*, the signal peptide sequence was removed by Abgent to increase the water solubility of the protein. Commercial human HDL was purchased from ProSpec‐Tany TechnoGene (Rehovot, Israel). ApoM^+^HDL and ApoM^−^HDL were separated by affinity chromatography, as previously described [2]. The total protein extraction kit and BCA protein assay kit were purchased from BestBio Biotechnology (Shanghai, China). Rapid Wright‐Giemsa staining solution was purchased from Sangon Biotech (Shanghai, China). ApoM monoclonal and β‐actin antibodies were purchased from Abnova (cat. no. H00055937‐M03, Taiwan, China) and Arigo Biolaboratories (cat. no. ARG62346, Taiwan, China), respectively. Antibodies against mouse CD11b (cat. no.101229) and F4/80 (cat. no.12321) were obtained from BioLegend (San Diego, CA, USA). LPS (cat. no. L4516) was purchased from Sigma‐Aldrich. IL‐4 (cat. no.214‐14) was purchased from PeproTech (Rocky Hill, NJ, USA). All reagents and solvents were of the highest grade available, and cell culture was tested.

### Animals

Male C57BL/6N mice with different genotypes but of identical age and body weight were used in this study (8–10 weeks old, 23–25 g). Wild‐type (WT) mice were obtained from the Center for Experimental Animals of Soochow University (Suzhou, China). ApoM‐deficient (*ApoM* ^−/−^) mice with C57BL/6N background were generated by homologous recombination with the help of the Model Animal Research Center of Nanjing University (Jiangsu, China) as previously described [21]. Chimeric male mice were identified using PCR and were bred with C57BL/6N female mice to generate F1 offsprings. The F1 offsprings were crossed for at least two to three generations until they were *ApoM* ^−/−^ homozygous. SR‐BI‐deficient (*SR‐BI* ^−/−^) mice with C57BL/6N background were generously provided by Nanjing Medical University. To obtain *ApoM/SR‐BI* DKO, male homozygous *ApoM^+/+^SR‐BI* ^−/−^ mice were crossbred with female homozygous *ApoM* ^−/−^ *SR‐BI^+/+^* mice. Littermates from that generation (*ApoM^+/−^SR‐BI^+/−^*) were then backcrossed to obtain *ApoM* ^−/−^ *SR‐BI* ^−/−^ mice. Because of the low reproduction rate in DKO mice, they were fed a 1% probucol chow diet to reduce blood lipids during the reproductive period. The fertility rate of mice increased significantly after medication. The genotypes of offsprings and laboratory mice were analyzed by PCR, as previously described [22, 23]. All mice were maintained under a 12:12‐h light–dark cycle in a temperature‐controlled facility (25 °C) with access to standard chow. All animal procedures were approved by the Animal Care and Use Committee of Soochow University, Suzhou, China (Permit Number: SYXK(Su)2017‐0043).

### Cell culture

The peritoneal macrophages were harvested from the peritoneal cavity of mice, as previously described [24]. Briefly, mice were injected intraperitoneally with 2 mL of 3% thioglycollate broth to recruiting a larger number of macrophages. After 3 days, the mice were euthanized, and the peritoneal cavities were washed with 5 mL of cold PBS. After a gentle massage of the abdominal wall, the peritoneal fluid containing macrophages was collected. After centrifugation for 5 min at 180 *g* and 4 °C, the cells were seeded into 48‐well plates (4 × 10^5^ cells/well) or 6‐well plates in RPMI 1640 medium supplemented with 10% FBS. The cells were allowed to adhere to the substrate by culturing for 2 h at 37 °C. Non‐adherent cells were removed by gently washing twice with warm PBS. Adherent cells were selected as macrophages and were maintained in culture for further use. The murine macrophage cell line Ana‐1 was purchased from the Shanghai Cell Bank of the Chinese Academy of Sciences, and the cells were cultured in regular tissue culture dishes in RPMI 1640 medium supplemented with 10% FBS at 37 °C in a humidified incubator with 5% CO_2_, and 95% air. The Ana‐1 cell line was established from bone marrow cells of C57BL/6 (H‐2^b^) mice infected with J2 recombinant retrovirus for immortalization [25].

### Wright staining and flow cytometry

The morphology of peritoneal macrophages was observed by microscopy after Wright staining, according to the manufacturer’s instructions. Quantification of murine peritoneal macrophages was performed by flow cytometry. Cell suspensions were processed for staining of macrophage surface markers, including F4/80 and CD11b. Data were collected using the BD FACSCanto II (BD Biosciences, San Jose, CA, USA) and analyzed using the flowjo software (Tree Star Inc. San Carlos, CA, USA).

### Phenotypic analysis of peritoneal macrophages

WT mice were induced with thioglycollate broth as described above. Control group was peritoneal injection with the same amount of normal sodium. Peritoneal macrophages from control group exposure to LPS (100 ng·mL^−1^) or IL‐4 (20 ng·mL^−1^) overnight for M1 or M2 polarization. The mRNA expressions of M1 marker iNOS and M2 marker Arg‐1 were examined by means of qRT‐PCR.

### Identification of PCR amplification products and capillary western immunoassay

The PCR product of *ApoM* was validated by agarose gel electrophoresis and sequencing at Sangon Biotech Co., Ltd. Capillary western immunoassay (Wes) was performed in protein lysates from Ana‐1 cell, peritoneal macrophages, liver, and kidney. Briefly, protein lysates were analyzed on a Wes system (ProteinSimple, Santa Clare, CA, USA) using a 12–230 kDa separation module (Bio‐Techne Ltd., Abingdon, UK). Level of ApoM (1 : 75) was normalized using the reference protein β‐actin (1 : 50). The peaks were analyzed using Compass software (ProteinSimple, Santa Clare, CA, USA). Detailed procedures are as described in the cited reference [26].

### Uptake of NBD‐cholesterol by Ana‐1 cells and mouse peritoneal macrophages

The NBD fluorophore can be attached to carbon‐22 or carbon‐25 on the side chains of cholesterol. The excitation and emission wavelength of NBD‐cholesterol are 469 and 537 nm, respectively. Different concentrations of NBD‐cholesterol and NBD (2.5–10 μm) were prepared before the experiments. Briefly, 2 mg of NBD‐cholesterol (molecular weight, 494.63) was dissolved in 4 mL of 37 °C absolute ethanol to obtain a concentration of 0.5 mg·mL^−1^ (1 × 10^3^ μm) NBD‐cholesterol. The dissolution of NBD was similar to that of NBD‐cholesterol. These solutions were mixed slowly into RPMI 1640 medium without phenol red to three final concentrations of 2.5, 5, and 10 μm. Ana‐1 cells were cultured as described above and seeded into 48‐well plates at a concentration of 2 × 10^5^ cells/well. Following activation by PMA (160 nm) for 24 h, cells were washed twice with PBS and incubated in serum‐free medium supplemented with NBD‐cholesterol (2.5–10 μm) at 37 °C. A similar experiment was performed using NBD. Then, the cells and culture supernatants were collected at 0, 0.5, 1, 2, 4, 6, 8, and 12 h. Peritoneal macrophages from the mice of four different genotypes were seeded into 48‐well plates (2 × 10^5^ cells/well). After incubation for 24 h, the peritoneal macrophages were loaded with 10 μm NBD‐cholesterol. Then, the cells and culture supernatants were collected at 1, 2, 4, 6, and 12 h. At each time point, the cells were washed twice with warm PBS and lysed with 1% Triton X‐100. The QuantiFluor™‐ST Handheld Fluorometer (Promega, Madison, WI, USA) was used to measure the fluorescence intensity of the supernatant and cell lysate. The uptake ratio was calculated as the percentage of NBD‐cholesterol in cells at a given time relative to the total NBD‐cholesterol content in the supernatant and cells.

### Expression analysis of cholesterol transport‐related genes

The mRNA levels of cholesterol transport‐related genes were analyzed by real‐time quantitative PCR. Peritoneal macrophages from the mice of four different genotypes were loaded with or without 10 μm NBD‐cholesterol for 6 h. Then, the total RNA from peritoneal macrophages was isolated according to the manufacturer’s protocol using the RNAeasy™ Animal RNA Isolation Kit with Spin Column (Beyotime Biotechnology, Shanghai, China). First‐strand cDNA was synthesized from 2 μg of total RNA using the RevertAid™ First‐Strand cDNA Synthesis Kit (Thermo Fisher Scientific, Waltham, MA, USA). primer premier 5.0 software (Premier Biosoft, Palo Alto, CA, USA) was used to design primers and probes (Table 1). Quantitative gene expression analysis of *Abca1*, *Abcg1*, *ApoA‐I*, *ApoE,* and the reference gene *gapdh* was performed by RT‐qPCR on the LightCycler480^®^II system (Roche, Basel, Switzerland).

**Table 1:** Sequences of primers and probes used in real‐time RT‐PCR (RT‐qPCR).

Gene	Primer / Probe	Sequence (5′–3′)
*Abca1*	Forward	GAAGTTTCTGCCCTCTGTGGT
	Reverse	CACATCTCATCTCCCGACCC
	Probe	TACCGAGGAAGAAGCTCGATGCAGC
*Abcg1*	Forward	ATGCTGCTGCCTCACCTCACT
	Reverse	GGAGAAGGATGAAGGCAGACG
	Probe	TCAGGAGGCCATGATGGTGTCCG
*ApoA‐I*	Forward	GGATGAAAGCTGTGGTGCTG
	Reverse	GGCACGTATGGCAGCAAGA
	Probe	CGTGGCTCTGGTCTTCCTGACAGG
*ApoE*	Forward	CCGTGCTGTTGGTCACATTG
	Reverse	CGAGTGGCAAAGCAACCAA
	Probe	TGACAGGATGCCTAGCCGAGGGAG
*gapdh*	Forward	TCTTGTGCAGTGCCAGCCT
	Reverse	TGAGGTCAATGAAGGGGTCG
	Probe	AGGTCGGTGTGAACGGATTTGGC
*ApoM*	Forward	GCTTTCTCCTCTACAATCGGTCAC
	Reverse	CGGGCAGGCCTCTTGATT
	Probe	ACCTCTTGCTTGGACTTCAAAGCCTTCTTA
*iNOS*	Forward	GGAGCGAGTTGTGGATTGTC
	Reverse	TCTCTGCCTATCCGTCTCGT
	Probe	TACACCACACCAAACTGTGTGCCTGGA
*Arg‐1*	Forward	TGCATATCTGCCAAAGACATCG
	Reverse	CTTCCATCACCTTGCCAATCC
	Probe	GTACATTGGCTTGCGAGACGTAGACCCT

### Efflux of NBD‐cholesterol from Ana‐1 cells

Before the efflux studies were conducted, the Ana‐1 cells were incubated for 4 h in 10 μm NBD‐cholesterol without serum. First, washed cells were incubated for 0, 0.5, 1, 2, 4, 6, and 8 h at 37 °C in serum‐free medium in the presence of ApoM^+^HDL (20 μg·mL^−1^), ApoM^−^HDL (20 μg·mL^−1^), or rh‐ApoM (10 μg·mL^−1^). The supernatant and cells were collected at each time point to measure fluorescence intensity. Second, washed cells were incubated with 10–100 μg·mL^−1^ rh‐ApoM, 20 μg·mL^−1^ ApoM^+^HDL (ApoM^−^HDL), or a combination of both rh‐ApoM and ApoM^+^HDL (ApoM^−^HDL). The supernatant and cells were collected after 4 h of induction. The efflux ratio was calculated as the percentage of NBD‐cholesterol released into the medium relative to the total NBD‐cholesterol content in the medium and cells. As a control, Ana‐1 cells were incubated with basic medium to obtain values for ‘background’ efflux.

### Statistical analysis

Each experiment is representative of at least three independent experiments. Data are shown as the mean ± SD. Statistical analysis was performed using two‐way ANOVA followed by Bonferroni test or one‐way ANOVA test. All calculations were performed using graphpad prism software (GraphPad Software, Inc., San Diego, CA, USA). Statistical significance was set at *P* < 0.05.

## Results

### Phenotype identification of thioglycollate‐induced peritoneal murine macrophages

**Fig. 1:**
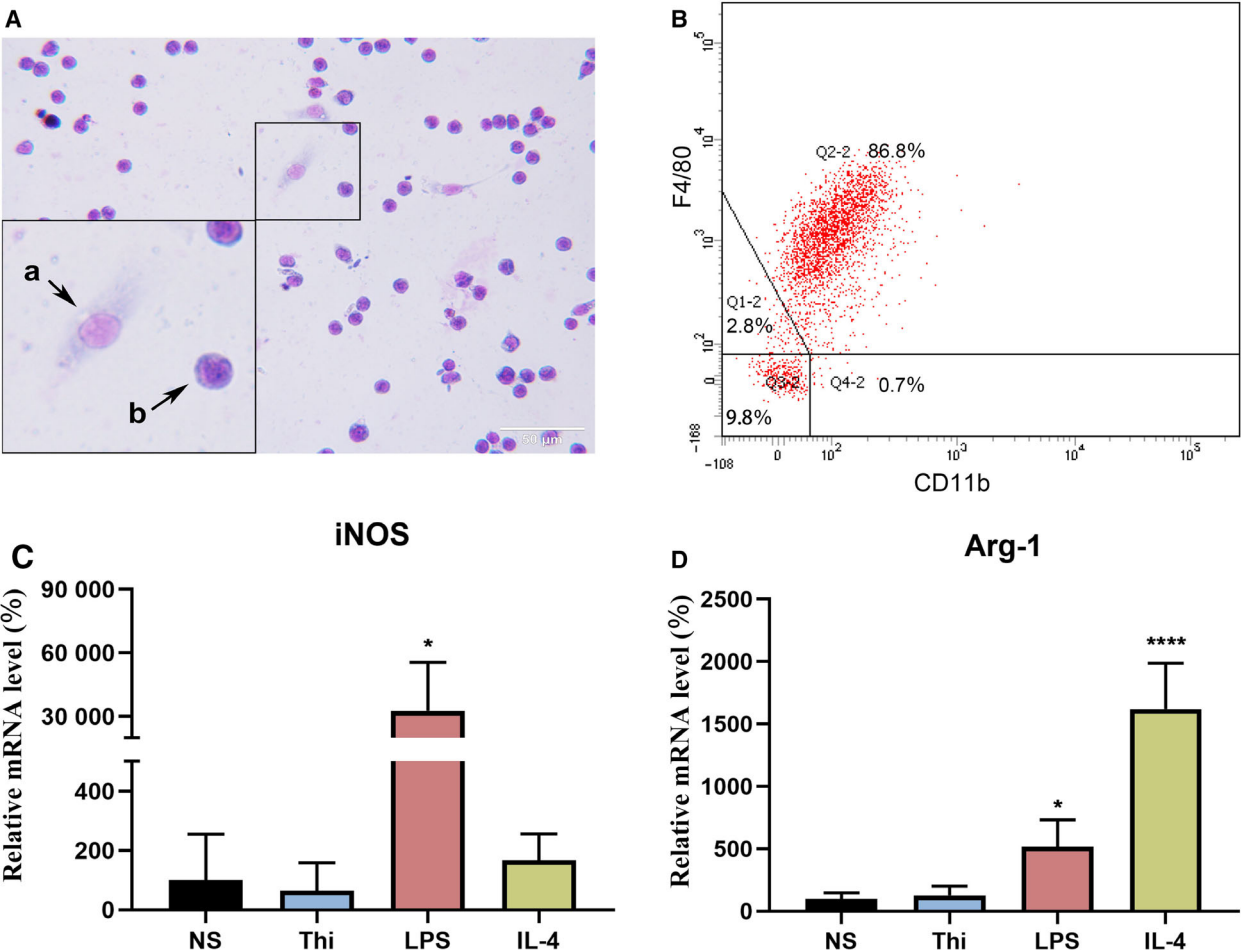
Phenotype identification of thioglycollate‐induced peritoneal murine macrophages. (A) Cell purity was monitored by Wright staining. a: fibroblast. b: macrophages. Scale bar: 50 μm. (B) Quantitative analysis of peritoneal murine macrophages by flow cytometry. (C, D) The mRNA expression level of M1 and M2 macrophage markers. Data were normalized to the NS group as 100%. Values are mean ± SD (*n* = 4 per group). Statistical analysis was performed using one‐way ANOVA test. NS: normal saline group; Thi: thioglycolate‐elicited group; LPS: LPS‐elicited group (M1, 100 ng·mL^−1^); IL‐4: IL‐4‐elicited group (M2, 20 ng·mL^−1^). (*P < 0.5, ****P < 0.001).

Figure 1A shows the purity of mouse peritoneal macrophages by Wright staining. As shown in Fig. 1A (b), predominant cells are round or oval macrophages with deeply stained nuclei and little cytoplasm. Occasionally, a few spindle fibroblasts with loose chromatin and abundant cytoplasm were observed in the field (Fig. 1A(a)). The original image is shown in Fig. S1. CD11b and F4/80 are macrophage surface markers. Flow cytometric analysis revealed that the percentage of F4/80^+^ CD11b^+^ cells was 86.8 % (Fig. 1B). Macrophages are typically divided into two groups based on whether they are classically (pro‐inflammatory, M1) or alternatively activated (anti‐inflammatory, M2). High levels of cellular induced nitric oxide synthase (iNOS) and type‐1 arginase (Arg‐1) are often observed in M1‐ and M2‐polarized macrophages. Thus, we quantified cellular iNOS and Arg‐1 by RT‐qPCR to evaluate macrophage polarization. Peritoneal murine macrophages derived from WT mice were divided into four groups and cultured for 12 h: normal saline group, thioglycolate‐elicited group, LPS‐elicited group (M1, 100 ng·mL^−1^), and IL‐4‐elicited group (M2, 20 ng·mL^−1^). As shown in Fig. 1C,D, the expressions of iNOS and Arg‐1 were higher than those in the other groups after LPS and IL‐4 stimulation. However, compared with the normal saline group, thioglycollate‐induced macrophages did not polarize.

### Expression of ApoM in macrophages, liver, and kidney

**Fig. 2:**
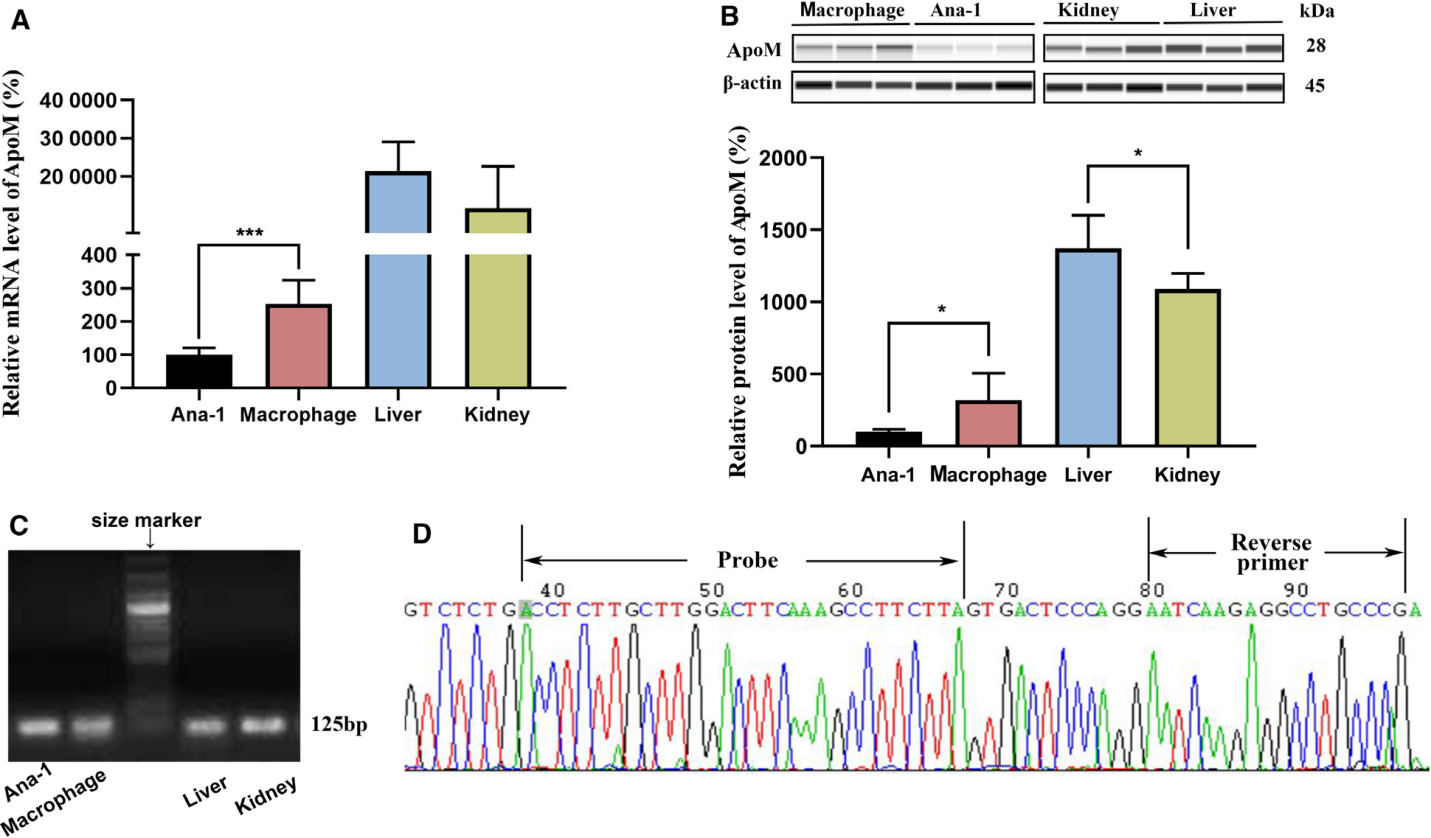
Expression of ApoM in Ana‐1 cells, peritoneal macrophages, liver, and kidney from WT mice. (A) The expression level of ApoM mRNA in Ana‐1 cells, peritoneal macrophages, liver, and kidney from WT mice. Data were normalized to the levels of ApoM in Ana‐1 cells as 100%. (***P < 0.01). (B) Capillary western immunoassay of ApoM protein in Ana‐1 cells, peritoneal macrophages, liver, and kidney from WT mice. Data were normalized to the levels of ApoM in Ana‐1 cells as 100%. (*P < 0.5). (C) Agarose gel electrophoresis of ApoM PCR product derived from Ana‐1 cells, peritoneal macrophages, liver, and kidney from WT mice. The middle lane contains DNA size markers. Lanes kidney and liver were the positive controls. (D) The sequencing of the PCR product in macrophages from WT mice. The PCR products were sequenced forward. Both probe and reverse‐primer sequences were matched with the products. Values are mean ± SD (*n* = 6 per group). Statistical analysis was performed using one‐way ANOVA test.

We characterized the expression of ApoM in Ana‐1 cells, peritoneal macrophages, hepatocytes, and kidney cells from WT mice. Fig. 2A shows the mRNA level of ApoM in Ana‐1, peritoneal macrophages, liver, and kidney. As shown in the RT‐qPCR analysis of mRNA expression levels, despite the much lower expression level compared with the liver and kidney, ApoM was expressed in both Ana‐1 and macrophages. The expression level of ApoM in peritoneal macrophages was ~ 2.5 times higher than that in Ana‐1 cells. Fig. 2B confirmed the protein level of ApoM by capillary western immunoassay. ApoM protein levels in macrophage, Ana‐1, kidney, and liver detected by western blot based on capillary electrophoresis technology and full‐length blots are presented in Fig. S2. Agarose gel electrophoresis and sequencing were subsequently performed, and the results confirmed that the PCR products were mouse ApoM (Fig. 2C,D). The original image of agarose gel electrophoresis is shown in Fig. S3.

### Uptake of NBD‐cholesterol in Ana‐1 cells

**Fig. 3:**
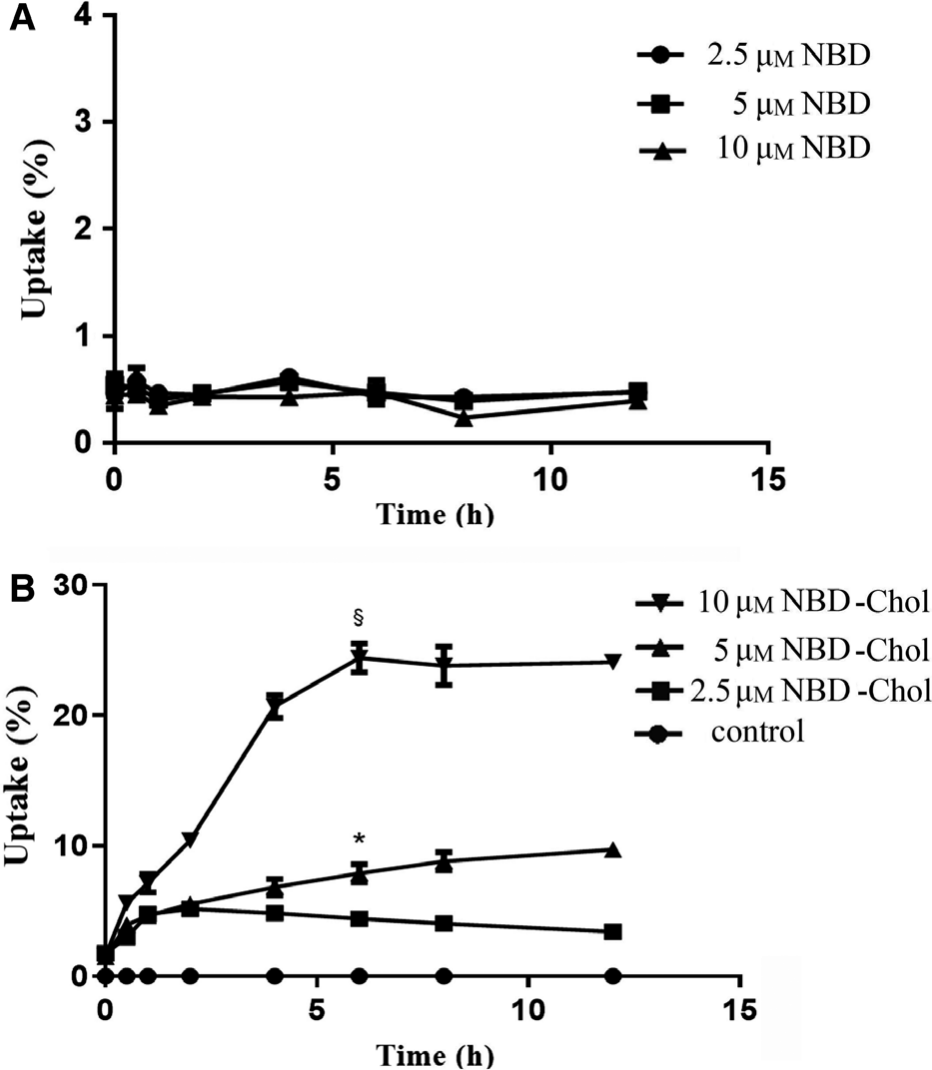
Uptake ratio of NBD‐cholesterol in cultured Ana‐1 cells. (A) Ana‐1 cells were incubated with NBD (2.5–10 μm) for 0, 0.5, 1, 2, 4, 6, 8, and 12 h. (B) Ana‐1 cells were incubated with or without (control) NBD‐cholesterol (2.5–10 μm) for 0, 0.5, 1, 2, 4, 6, 8, and 12 h. The fluorescence intensity of the supernatant and cell lysate was measured at each time point to calculate the uptake ratio. Values are the mean ± SD (*n* = 3 per group and three independent experiments). Statistical analysis was performed using two‐way ANOVA followed by Bonferroni test. *Significantly different from 2.5 μm NBD‐Chol (*P* < 0.05). § significantly different from 5μM NBD‐Chol (*P* < 0.05). uptake (%) = fluorescence intensities of the cell lysate / (fluorescence intensities of supernatant + fluorescence intensities of the cell lysate) × 100%.

To ensure that the fluorescence intensity of NBD represents cholesterol content, we first ruled out the possibility of NBD uptake by cells. The results showed that the uptake of NBD by Ana‐1 cells was independent of time and concentration (Fig. 3A); however, the uptake of NBD‐cholesterol was concentration‐dependent (Fig. 3B). In each group, the uptake ratio significantly increased after incubation with NBD‐cholesterol for 30 min (*P* < 0.05). When the cells were incubated with 10 μm NBD‐cholesterol, the uptake ratios increased over time in a certain range and plateaued at 6 h (Fig. 3B). There was no significant difference in the uptake ratios at 8 and 6 h (*P* > 0.05). The curve was most stable after 6 h, so we chose 10 μm NBD‐cholesterol as the optimal concentration for follow‐up experiments.

### Deficiency of ApoM and SR‐BI impairs the uptake of NBD‐cholesterol in peritoneal macrophages

**Fig. 4:**
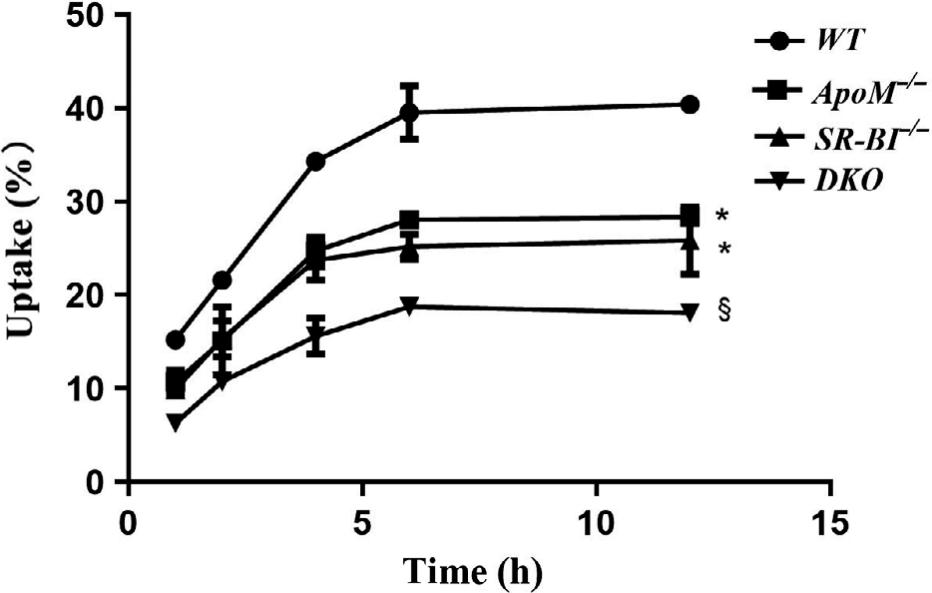
Uptake ratio of NBD‐cholesterol in peritoneal macrophages. Peritoneal macrophages were incubated in serum‐free medium supplemented with 10 μm NBD‐cholesterol for 1, 2, 4, 6, and 12 h. The fluorescence intensity of the supernatant and cell lysate was measured at each time point to calculate the uptake ratio. Values are the mean ± SD (*n* = 3 per group and three independent experiments). Statistical analysis was performed using two‐way ANOVA followed by Bonferroni test. *Significantly different from peritoneal macrophages of wild‐type mice starting at 1 h (*P* < 0.05). § significantly different from peritoneal macrophages of *SR‐BI* ^−/−^ mice starting at 1 h (*P* < 0.05). uptake (%) = fluorescence intensities of the cell lysate/(fluorescence intensities of supernatant + fluorescence intensities of the cell lysate) × 100%.

Peritoneal macrophages from WT, *ApoM* ^−/−^, *SR‐BI* ^−/−^ and DKO were used to study the uptake ratios of NBD‐cholesterol. The data are presented in Fig. 4. The uptake ratios of all the groups plateaued at 6 h. There was no significant difference between the uptake ratios at 6 and 12 h (*P* > 0.05). However, at the plateau stage, NBD‐cholesterol uptake by *ApoM* ^−/−^ mouse macrophages was significantly decreased compared to that by WT mouse macrophages (*P* < 0.05). Additionally, peritoneal macrophages from DKO mice also showed a lower uptake ratio than *SR‐BI* ^−/−^ mice and *ApoM* ^−/−^ mice (*P* < 0.05).

**Fig. 5:**
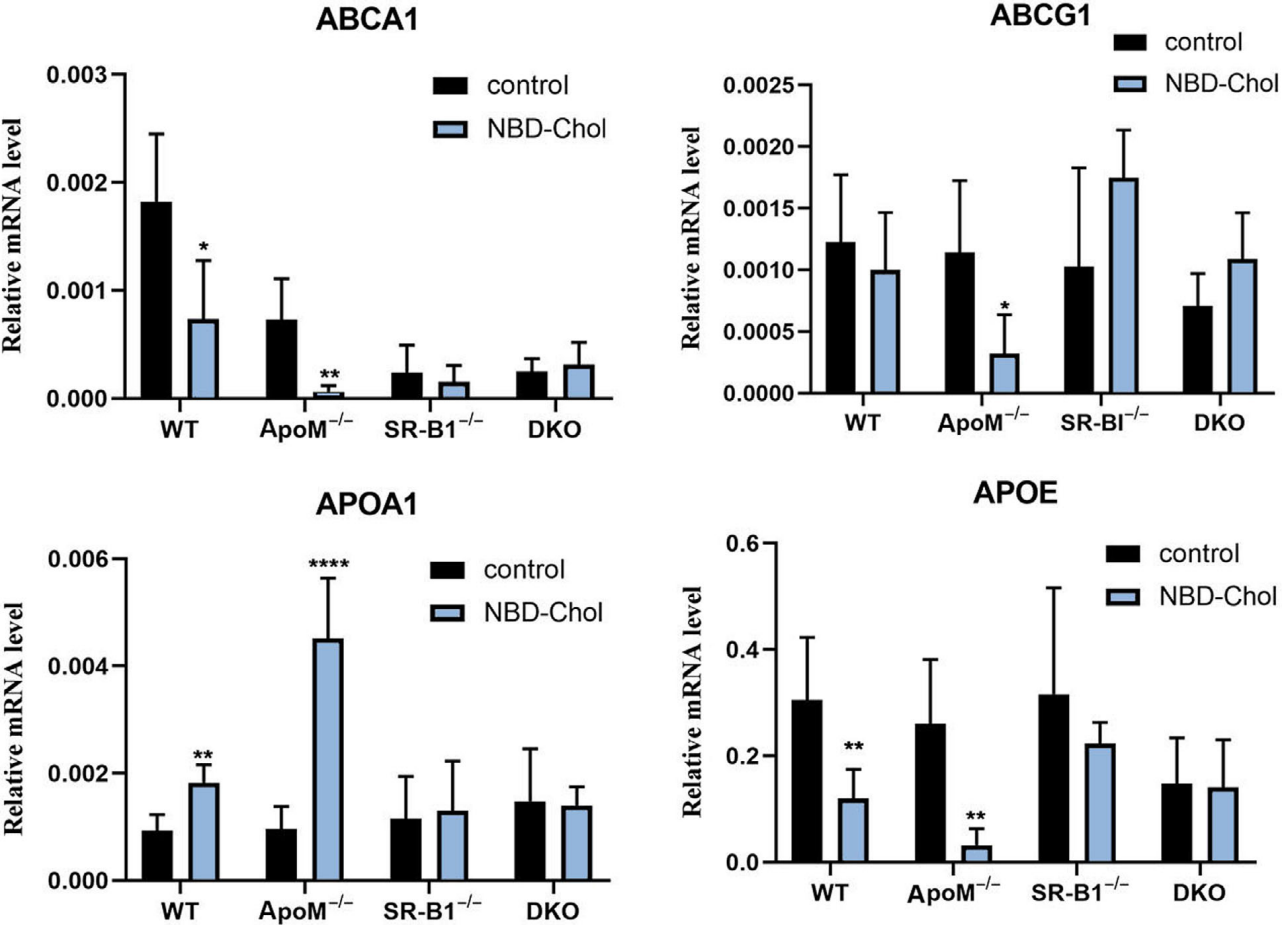
Gene expression of ABCA1, ABCG1, ApoA‐I, and ApoE in peritoneal macrophages. Peritoneal macrophages were cultured in the presence and absence of 10 μm NBD‐cholesterol for 6 h. Control: Peritoneal macrophages were cultured in NBD‐cholesterol‐free medium for 6 h. NBD‐C: Peritoneal macrophages were cultured in NBD‐cholesterol‐contain medium for 6 h. Gene expression was analyzed by RT‐qPCR. Values are the mean ± SD (*n* = 4 or 5 per group). Statistical analysis was performed using the paired‐samples *t* test. *Significantly different from the control (*P* < 0.05). **Significantly different from the control (*P* < 0.01). ****Significantly different from the control (*P* < 0.0001).

In addition, we detected the mRNA levels of *ABCA1*, *ABCG1*, *ApoA‐I,* and *ApoE* in these macrophages 6 h after incubation with or without NBD‐cholesterol. As shown in Fig. 5, the *ABCA1* levels were lower in peritoneal macrophages from *ApoM* ^−/−^ *, SR‐BI* ^−/−^, and DKO mice than in those from WT mice. In the WT and *ApoM* ^−/−^ groups, the mRNA expression of *ABCA1* and *ApoE* decreased significantly after cholesterol ingestion, while the expression of *ApoA‐I* increased significantly. The expression of *ABCG1* decreased only in the *ApoM* ^−/−^ group after cholesterol ingestion.

### Effects of ApoM on the efflux of NBD‐cholesterol from Ana‐1 cells

**Fig. 6:**
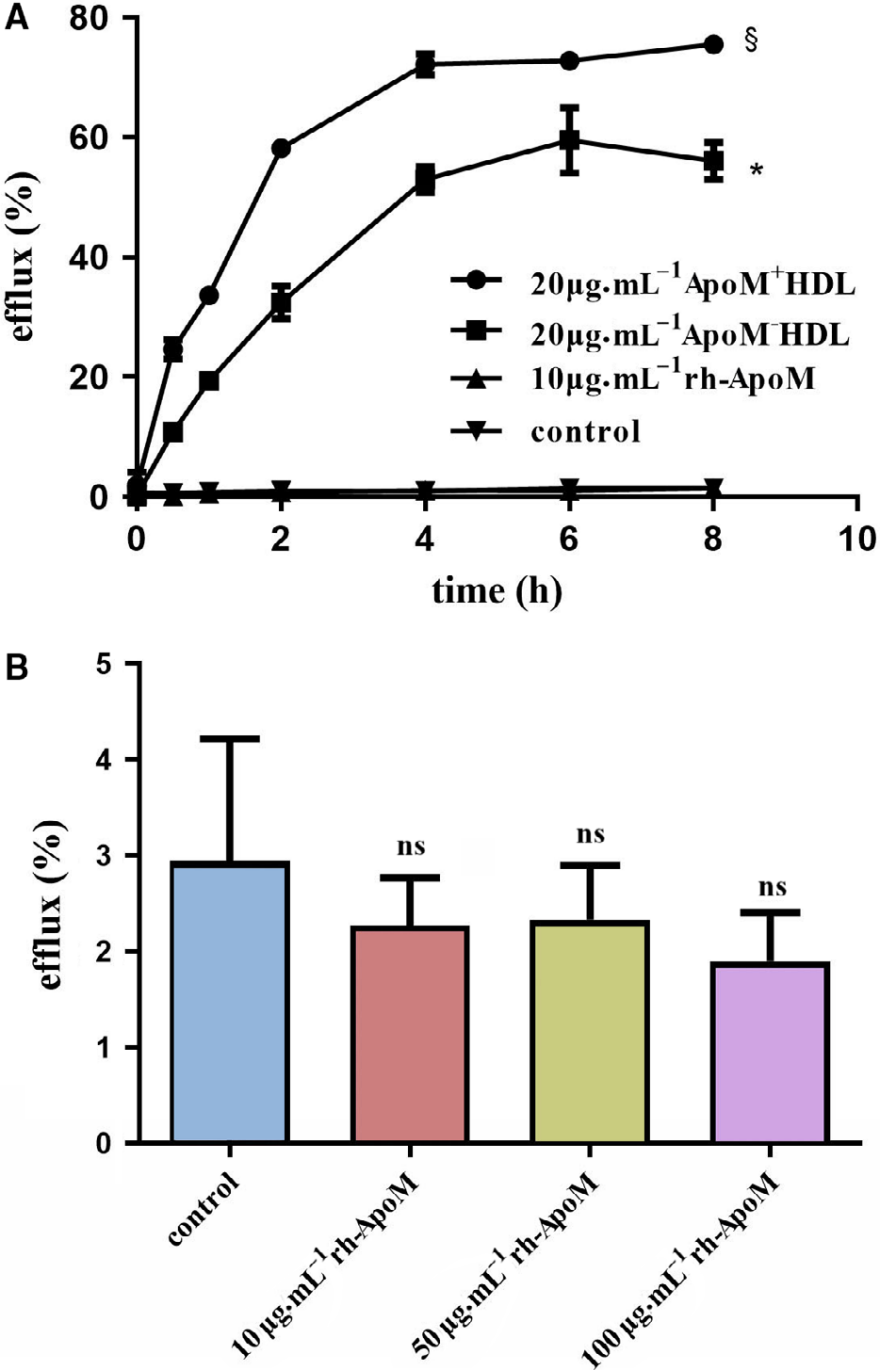
Cholesterol efflux from Ana‐1 cells with different ApoM‐inducing solutions. Ana‐1 cells were incubated for 4 h in serum‐free medium supplemented with 10 μm NBD‐cholesterol. (A) The labeled cells were incubated with 20 μg·mL^−1^ ApoM^+^HDL, 20 μg·mL^−1^ ApoM^−^HDL, or 10 μg·mL^−1^ rh‐ApoM for another 0–8 h. (B) The labeled cells were incubated with 10–100 μg·mL^−1^ rh‐ApoM for 4 h. A set of cells was incubated with serum‐free medium as a control. Values are presented as the mean ± SD (*n* = 3–6 per group). Statistical analysis was performed using two‐way ANOVA followed by Bonferroni test and one‐way ANOVA test. *Significantly different from the control *(P* < 0.05), § Significantly different from ApoM^−^HDL (*P* < 0.05). *ns* not significantly different from the control (*P* > 0.05). efflux (%) = fluorescence intensities of supernatant / (fluorescence intensities of supernatant + fluorescence intensities of the cell lysate) × 100%.

**Fig. 7:**
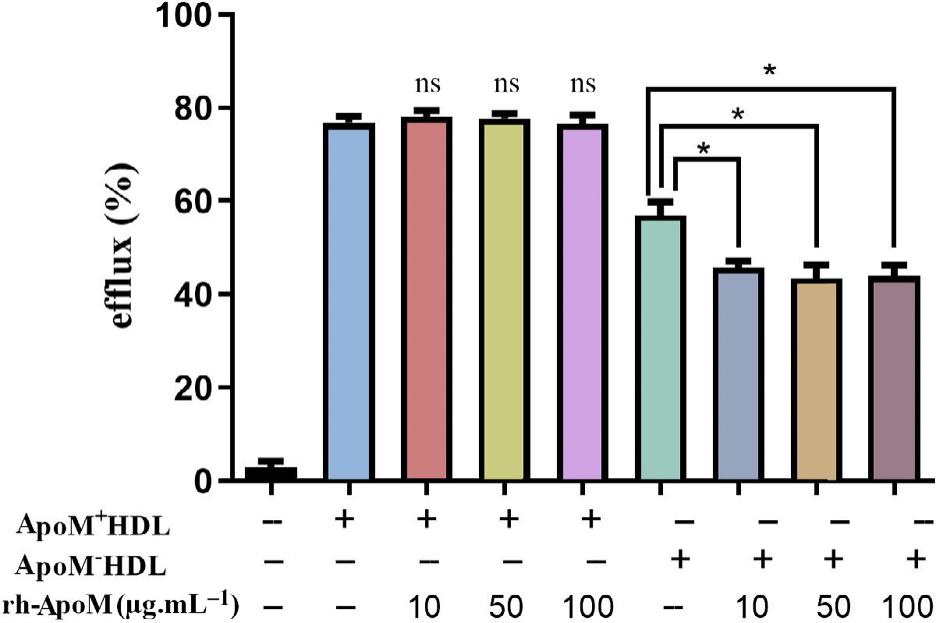
Effects of rh‐ApoM on cholesterol efflux from Ana‐1 cells. Ana‐1 cells were incubated with 10 μm NBD‐cholesterol for 4 h. A concentration of 20 μg·mL^−1^ ApoM^+^HDL (ApoM^−^HDL) or a combination of both ApoM^+^HDL (ApoM^−^HDL) and rh‐ApoM was used to induce cholesterol efflux. A set of cells was incubated with serum‐free medium as a control. Values are mean ± SD (*n* = 6 per group). Statistical analysis was performed using one‐way ANOVA test. *P < 0.001 vs. ApoM^−^HDL. *ns* not significantly different from ApoM^+^HDL (*P* > 0.05). efflux (%) = fluorescence intensities of supernatant/(fluorescence intensities of supernatant + fluorescence intensities of the cell lysate) × 100%.

Fig. 6A shows the effects of ApoM on cholesterol efflux. ApoM^+^HDL (20 μg·mL^−1^) and ApoM^−^HDL (20 μg·mL^−1^) enhanced cholesterol efflux from Ana‐1 cells, and ApoM^+^HDL seemed to have a stronger effect on promoting cholesterol efflux than ApoM^−^HDL (*P* < 0.05). The efflux ratio of NBD‐cholesterol increased over time, peaking at 4 h, and subsequently remained constant (Fig. 6A). There was no detectable difference in the cholesterol efflux‐stimulating capacity between the control and rh‐ApoM (10 μg·mL^−1^). It was supposed that the concentration of rh‐ApoM was too low, so we increased its concentration to 50 and 100 μg·mL^−1^. However, the result showed that increasing rh‐ApoM concentration did not promote cholesterol efflux (Fig. 6B). Figure 7 showed that rh‐ApoM (10 μg·mL^−1^) did not change the ApoM^+^HDL‐induced cholesterol efflux, but it reduced the effects of ApoM^−^HDL on cholesterol efflux (*P* < 0.05).

## Discussion

In this study, we mainly explored the role of ApoM in two aspects: the uptake and efflux of cholesterol in mouse macrophages. In the investigation of ApoM knockout mice, we found that similar to SR‐BI, ApoM deficiency leads to impairment of cholesterol uptake in macrophages. ApoM^+^HDL facilitated more cholesterol efflux from Ana‐1 cells than ApoM^−^HDL. Rh‐ApoM alone did not affect the cholesterol efflux from macrophages. In contrast, when working together with ApoM^−^HDL, the ApoM^−^HDL‐induced cholesterol efflux decreased.

The formation of excess cholesterol deposits in macrophages is the pathological basis for the development of atherosclerosis [27]. RCT is the process by which excess cholesterol in peripheral tissues is transported to the liver via HDL [28]. The efflux of cholesterol from lipid‐loaded macrophages is not only the initial step of RCT but also the limiting step [29]. Regulation of cholesterol homeostasis is highly likely to be an effective treatment for atherosclerosis; therefore, it is necessary to study the factors that influence cholesterol transport. The fluorescence intensity of NBD‐cholesterol is proportional to the concentration of cholesterol [30, 31]. When cells were treated with NBD fluorescent dye alone, almost no fluorescence (less than 1%) was observed in the cells; however, an obvious fluorescence gradient was detected in cells cultured with different concentrations of NBD‐cholesterol. Therefore, we assumed that the NBD fluorescent dye was transported into cells along with cholesterol, and the fluorescence intensity detected in the following experiments represented the cholesterol content. In this study, the uptake ratio of 10 μm NBD‐cholesterol by Ana‐1 cells and peritoneal macrophages plateaued at 6 h which is consistent with the results of a previous study [32]. We chose 10 μm as the working concentration for better results in subsequent experiments, given that Ana‐1 cells uptake cholesterol in a concentration‐dependent manner. Interestingly, at low NBD‐cholesterol concentration such as 2.5 μm, the uptake of cholesterol decreases over time. The balance between hydrolysis and esterification of cholesterol plays a key role in maintaining cholesterol homeostasis and preventing the generation of foam cells [33]. When cells are stimulated with low concentrations of cholesterol, the cholesterol intake is still within a range that the cells can manage. Cells process and metabolize cholesterol over time; therefore, the amount of cholesterol decreases. In the case of cholesterol overload, macrophages can be transformed into foam cells. Cholesterol accumulates in the cell beyond the metabolic capacity of the cell, and the amount of metabolized cholesterol is negligible compared to that taken in.

ApoM is a member of the lipocalin superfamily, which has different functions in lipid construction and transport [34]. It was reported that SNPs in the proximal promoter region of the *ApoM* gene are associated with dyslipidemia [35]. *In vivo*, the concentration of ApoM in the plasma is positively correlated with total cholesterol [36]. These studies suggest that ApoM might be associated with cholesterol metabolism.

Previous studies have shown that SR‐BI plays an important role in facilitating cholesterol trafficking [37]. SR‐BI deficiency impairs both lipid influx and efflux from cells [38], which was verified in our experiments. Our previous work showed that SR‐BI deficiency also promotes ApoM expression [39] in liver cells, which indicates that they can compensate for each other *in vivo*. However, *in vitro*, increased ApoM might be independent of HDL‐mediated cholesterol uptake [39]. To explore whether ApoM affected cholesterol uptake, we tested the cholesterol uptake ability of macrophages derived from ApoM knockout mice. The results showed that the uptake of NBD‐cholesterol in peritoneal macrophages from *ApoM* ^−/−^ mice was lower than that in macrophages from WT mice. The same results were observed when comparing DKO mice to *SR‐BI* ^−/−^ mice, indicating that the deletion of ApoM might decrease cholesterol uptake, similar to SR‐BI deletion in peritoneal macrophages. Therefore, we believe that ApoM and SR‐BI might have synergistic effects on cholesterol uptake. Previous studies have generally focused on the effects of ApoM on HDL, but our results demonstrate for the first time that ApoM expressed in macrophages has the same function as SR‐BI in regulating cholesterol transport. On the one hand, this finding encouraged us to explore other physiological functions of ApoM in different cells and tissues. On the other hand, anti‐atherosclerotic therapies should perhaps not simply focus on increasing or decreasing the concentration of cholesterol transport‐related factors but also on maintaining intracellular cholesterol homeostasis. Cluster of differentiation 36 (CD36), a class B scavenger receptor similar to SR‐BI, proposed to be a crucial molecule in cholesterol homeostasis in various mechanisms including absorption/reabsorption, synthesis, and transport of cholesterol and bile acids. CD36 plays a crucial role in foam cell formation and LDL uptake in macrophages [40]. Animal studies performed on CD36‐deficient mice suggest that deficiency of CD36 prevents the development of atherosclerosis [41]. However, ApoM deficiency leads to lipid deposition and metabolic distress and promotes atherosclerosis [42]. Therefore, although ApoM affects macrophage cholesterol uptake like CD36, its function *in vivo* should be more complex.

At the same time, we detected the mRNA levels of cholesterol transport‐related factors among different genotypes with or without NBD‐cholesterol incubation. ABCA1 and ABCG1 govern the rate‐limiting step for cholesterol efflux from peripheral cells. In our study, ABCA1 levels were lower in peritoneal macrophages from *ApoM* ^−/−^ *, SR‐BI* ^−/−^, and DKO mice than in those from WT mice, indicating that the knockout of ApoM or SR‐BI could affect the expression of ABCA1. In the WT and *ApoM* ^−/−^ groups, the mRNA expression of ABCA1 and ApoE was decreased significantly after cholesterol ingestion, while the expression of ApoA‐I was increased significantly. The hepatic ApoM expression level in ApoE‐deficient mice was lower than that in WT mice. ApoE plays a role in accelerating the clearance of plasma ApoM [43]. Our results suggest that ApoM deficiency may also influence ApoE expression during macrophage cholesterol uptake. The expression of ABCG1 decreased only in the *ApoM* ^−/−^ group after cholesterol ingestion. This phenomenon is worthy of attention because cholesterol loading significantly decreases ABCA1 levels. It is well accepted that ABCA1‐mediated cholesterol efflux is initiated by the interaction of lipid‐ and cholesterol‐free ApoA‐I particles with ABCA1 transporters in peripheral tissues [44]. The expression level of ABCA1 is positively correlated with the capacity to promote cellular cholesterol efflux [45]. Sparrow *et al*. [46] reported that Ac‐LDL‐loading increases ABCA1 mRNA levels in macrophages. Venkateswaran *et al*. [47] reported that the degree of induction of ABCA1 mRNA was clearly dependent on the type of exogenously added lipid. Acetylated low‐density lipoprotein (Ac‐LDL), oxidized low‐density lipoprotein (ox‐LDL), and 25‐dihydroxycholesterol (25‐OHC) were more potent inducers of ABCA1 mRNA levels than LDL. Our results seemed inconsistent with those of previous studies. One possible explanation is that no exogenous lipid‐poor ApoA‐I as a cholesterol receptor in the supernatant was formed, so intracellular cholesterol did not efflux outside the cell (it can be verified by the control group in Fig 6). The effect of free cholesterol on the level of ABCA1 in macrophages is not clear. Another explanation is that in the WT group, the rate of cholesterol influx becomes too high and mechanisms of cholesterol homeostasis regulation do not function properly, which can lead to endoplasmic reticulum (ER) stress initiation. One consequence of ER stress is the downregulation of ABCA1, which leads to foam cell formation [48, 49]. From the current data, we can see that ApoM deletion affects the expression of cholesterol transport‐related genes in the process of cholesterol uptake. Further studies are required to elucidate the underlying mechanism.

To investigate whether rh‐ApoM, ApoM^+^HDL, and ApoM^−^HDL have different effects on cholesterol efflux, Ana‐1 cells loaded with NBD‐cholesterol were incubated with the above three particles. The data showed that ApoM^+^HDL particles were more efficient than ApoM^−^HDL in stimulating cholesterol efflux from Ana‐1 cells and reducing total levels of intracellular cholesterol, which is consistent with observations reported by Elsoe *et al* [5]. Kober reported that cholesterol efflux to HDL increased with increasing ApoM molecule content per HDL particles [8]. Their results and ours suggested that ApoM could increase the capacity of HDL to facilitate the transport of cholesterol to the extracellular acceptor.

**Fig. 8:**
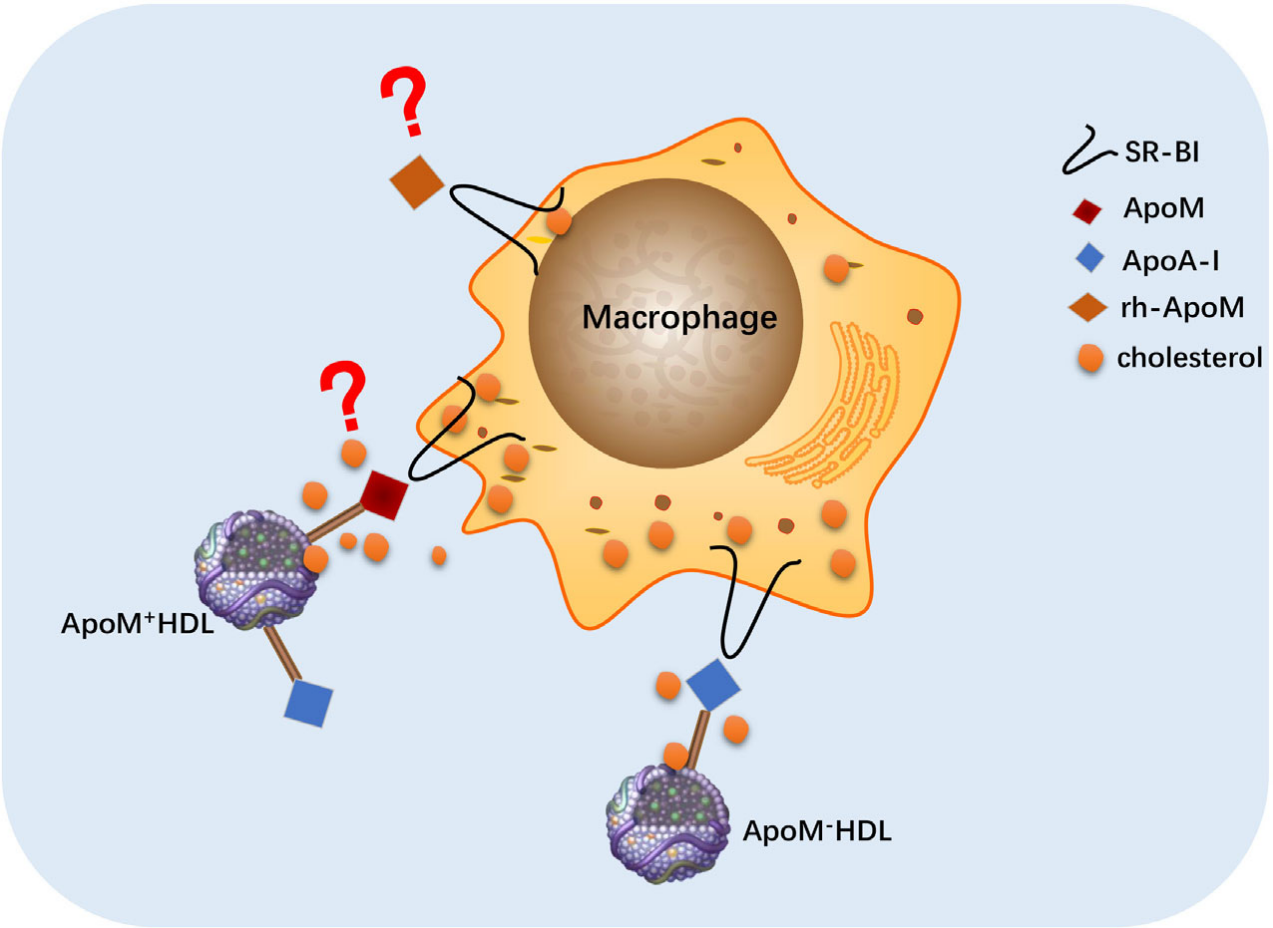
A hypothetic scheme illustrating a possible mechanism of how rh‐ApoM impairs the function of ApoM^−^HDL in promoting cholesterol efflux.

However, different concentrations of rh‐ApoM could not effectively promote cholesterol efflux from Ana‐1 cells. This result suggests that rh‐ApoM fails to promote cholesterol efflux while lacking HDL *in vitro*. To determine whether rh‐ApoM forms an effective complex with HDL to promote cholesterol efflux, we added rh‐ApoM to the supernatant containing ApoM^+^HDL or ApoM^−^HDL to observe the effects of rh‐ApoM on HDL‐mediated cholesterol efflux. The results showed that rh‐ApoM did not affect ApoM^+^HDL‐induced cholesterol efflux, whereas it significantly decreased ApoM^−^HDL‐induced cholesterol efflux. This indicates that *in vitro*, rh‐ApoM cannot bind ApoM^−^HDL to form effective ApoM^+^HDL complexes. To explain our conjecture more clearly, we drew a simple sketch map to illustrate (Fig. 8). Since both of ApoM^+^HDL and ApoM^−^HDL contain ApoA‐I, we hypothesized that the affinity of rh‐ApoM for SR‐BI was higher than that of ApoA‐I but lower than that of ApoM^+^HDL (i.e., ApoM^+^HDL > rh‐ApoM > ApoA‐I). When macrophages were incubated with rh‐ApoM and ApoM^−^HDL, although rh‐ApoM might compete with ApoM^−^HDL in combination with part of SR‐BI expressed on macrophages, it fails to promote cholesterol efflux while lacking HDL. Therefore, blocking the receptor that binds to ApoA‐I on ApoM^−^HDL will reduce ApoM^−^HDL‐induced cholesterol efflux. However, rh‐ApoM could not compete with ApoM^+^HDL, so it did not affect ApoM^+^HDL‐mediated cholesterol efflux. If ApoM is compared to a locomotive, HDL can be considered as a carriage. Cellular cholesterol cannot be transported by the ‘locomotive’ without the ‘carriage’. Taken together, these findings suggest that HDL promotes cholesterol efflux not only through the ApoA‐I pathway but also via the ApoM pathway, which hypothetically involves SR‐BI. Moreover, receptors on Ana‐1 cells may have a stronger affinity for rh‐ApoM than other ligands on ApoM^−^HDL, such as ApoA‐I, which results in an impaired ability of ApoM^−^HDL to promote cholesterol efflux. As shown in Fig. 8, there is no evidence of a direct interaction between ApoM and SR‐BI. However, this does not rule out the possibility that they work in concert in HDL‐dependent cholesterol efflux, which will be appealing to investigate in future studies.

In summary, our data suggest the importance of ApoM in the uptake and efflux of cholesterol in mouse macrophages. ApoM deficiency leads to decreased cholesterol uptake by mouse peritoneal macrophages. ApoM^+^HDL promotes cellular cholesterol efflux more efficiently than ApoM^−^HDL does.

## Conflict of interest

The authors declare no conflicts of interest.

## Author contributions

ZL, LGH, and XN conceived and supervised the study. YS, ZL, and LGH designed the experiments. YS, ZF, YY, and ZYX collected and analyzed the data. YS and ZF wrote the manuscript. All authors read and approved the final manuscript submitted for publication.

## Supporting information


**Fig. S1**. Wright staining of peritoneal macrophages from WT mice. Scale bar: 50 μm.
**Fig. S2**. ApoM protein levels in macrophage, Ana‐1, Kidney, and liver detected by western blot based on capillary electrophoresis technology. A pack with 25 capillary cartridges was used in this study. ApoM and β‐actin protein levels were detected by 12–230 kDa pre‐filled plates, respectively. The blots in Fig. 2B in main text are from blots which are framed.
**Fig. S3**. Agarose gel electrophoresis of apoM PCR product derived from Ana‐1 cells, peritoneal macrophages, liver, and kidney from WT mice. A1‐A6: Ana‐1. M1‐M6: Macrophage. L1‐L6: Liver. K1‐K6: Kidney. The blots in green box were used in Fig. 2C.

## Data Availability

The datasets used and/or analyzed during this study are available from the corresponding author on reasonable request.

## References

[1] Xu N and Dahlback B (1999) A novel human apolipoprotein (apoM). J Biol Chem 274, 31286–31290. 10531326 10.1074/jbc.274.44.31286

[2] Christoffersen C , Nielsen LB , Axler O , Andersson A , Johnsen AH and Dahlback B (2006) Isolation and characterization of human apolipoprotein M‐containing lipoproteins. J Lipid Res 47, 1833–1843. 16682745 10.1194/jlr.M600055-JLR200

[3] Christoffersen C , Obinata H , Kumaraswamy SB , Galvani S , Ahnstrom J , Sevvana M , Egerer‐Sieber C , Muller YA , Hla T , Nielsen LB *et al*. (2011) Endothelium‐protective sphingosine‐1‐phosphate provided by HDL‐associated apolipoprotein M. Proc Natl Acad Sci USA 108, 9613–9618. 21606363 10.1073/pnas.1103187108 PMC3111292

[4] Christoffersen C , Jauhiainen M , Moser M , Porse B , Ehnholm C , Boesl M , Dahlback B and Nielsen LB (2008) Effect of apolipoprotein M on high density lipoprotein metabolism and atherosclerosis in low density lipoprotein receptor knock‐out mice. J Biol Chem 283, 1839–1847. 18006500 10.1074/jbc.M704576200

[5] Elsoe S , Christoffersen C , Luchoomun J , Turner S and Nielsen LB (2013) Apolipoprotein M promotes mobilization of cellular cholesterol *in vivo* . Biochim Biophys Acta 1831, 1287–1292. 24046869 10.1016/j.bbalip.2013.04.009

[6] Wolfrum C , Poy MN and Stoffel M (2005) Apolipoprotein M is required for prebeta‐HDL formation and cholesterol efflux to HDL and protects against atherosclerosis. Nat Med 11, 418–422. 15793583 10.1038/nm1211

[7] Elsoe S , Ahnstrom J , Christoffersen C , Hoofnagle AN , Plomgaard P , Heinecke JW , Binder CJ , Bjorkbacka H , Dahlback B and Nielsen LB (2012) Apolipoprotein M binds oxidized phospholipids and increases the antioxidant effect of HDL. Atherosclerosis 221, 91–97. 22204862 10.1016/j.atherosclerosis.2011.11.031

[8] Kober AC , Manavalan APC , Tam‐Amersdorfer C , Holmer A , Saeed A , Fanaee‐Danesh E , Zandl M , Albrecher NM , Bjorkhem I , Kostner GM *et al*. (2017) Implications of cerebrovascular ATP‐binding cassette transporter G1 (ABCG1) and apolipoprotein M in cholesterol transport at the blood‐brain barrier. Biochim Biophys Acta Mol Cell Biol Lipids 1862, 573–588. 28315462 10.1016/j.bbalip.2017.03.003

[9] Acton S , Rigotti A , Landschulz KT , Xu S , Hobbs HH and Krieger M (1996) Identification of scavenger receptor SR‐BI as a high density lipoprotein receptor. Science 271, 518–520. 8560269 10.1126/science.271.5248.518

[10] Ji Y , Jian B , Wang N , Sun Y , Moya ML , Phillips MC , Rothblat GH , Swaney JB and Tall AR (1997) Scavenger receptor BI promotes high density lipoprotein‐mediated cellular cholesterol efflux. J Biol Chem 272, 20982–20985. 9261096 10.1074/jbc.272.34.20982

[11] Jian B , De La Llera‐Moya M , Ji Y , Wang N , Phillips MC , Swaney JB , Tall AR and Rothblat GH (1998) Scavenger receptor class B type I as a mediator of cellular cholesterol efflux to lipoproteins and phospholipid acceptors. J Biol Chem 273, 5599–5606. 9488688 10.1074/jbc.273.10.5599

[12] Linton MF , Tao H , Linton EF and Yancey PG (2017) SR‐BI: a multifunctional receptor in cholesterol homeostasis and atherosclerosis. Trends Endocrinol Metab 28, 461–472. 28259375 10.1016/j.tem.2017.02.001 PMC5438771

[13] Pagler TA , Rhode S , Neuhofer A , Laggner H , Strobl W , Hinterndorfer C , Volf I , Pavelka M , Eckhardt ER , Van Der Westhuyzen DR *et al*. (2006) SR‐BI‐mediated high density lipoprotein (HDL) endocytosis leads to HDL resecretion facilitating cholesterol efflux. J Biol Chem 281, 11193–11204. 16488891 10.1074/jbc.M510261200

[14] Fuentes M , Santander N and Cortes V (2018) Insulin increases cholesterol uptake, lipid droplet content, and apolipoprotein B secretion in CaCo‐2 cells by upregulating SR‐BI via a PI3K, AKT, and mTOR‐dependent pathway. J Cell Biochem 120, 1550–1559. 10.1002/jcb.27410 30278109

[15] Ji A , Meyer JM , Cai L , Akinmusire A , De Beer MC , Webb NR and Van Der Westhuyzen DR (2011) Scavenger receptor SR‐BI in macrophage lipid metabolism. Atherosclerosis 217, 106–112. 21481393 10.1016/j.atherosclerosis.2011.03.017 PMC3139003

[16] Adorni MP , Zimetti F , Billheimer JT , Wang N , Rader DJ , Phillips MC and Rothblat GH (2007) The roles of different pathways in the release of cholesterol from macrophages. J Lipid Res 48, 2453–2462. 17761631 10.1194/jlr.M700274-JLR200

[17] Marcel YL , Ouimet M and Wang MD (2008) Regulation of cholesterol efflux from macrophages. Curr Opin Lipidol 19, 455–461. 18769226 10.1097/MOL.0b013e32830f4a1d

[18] Jin X , Freeman SR , Vaisman B , Liu Y , Chang J , Varsano N , Addadi L , Remaley A and Kruth HS (2015) ABCA1 contributes to macrophage deposition of extracellular cholesterol. J Lipid Res 56, 1720–1726. 26203076 10.1194/jlr.M060053 PMC4548776

[19] Vedhachalam C , Duong PT , Nickel M , Nguyen D , Dhanasekaran P , Saito H , Rothblat GH , Lund‐Katz S and Phillips MC (2007) Mechanism of ATP‐binding cassette transporter A1‐mediated cellular lipid efflux to apolipoprotein A‐I and formation of high density lipoprotein particles. J Biol Chem 282, 25123–25130. 17604270 10.1074/jbc.M704590200

[20] Wang N , Lan D , Chen W , Matsuura F and Tall AR (2004) ATP‐binding cassette transporters G1 and G4 mediate cellular cholesterol efflux to high‐density lipoproteins. Proc Natl Acad Sci USA 101, 9774–9779. 15210959 10.1073/pnas.0403506101 PMC470750

[21] Wang Z , Luo G , Feng Y , Zheng L , Liu H , Liang Y , Liu Z , Shao P , Berggren‐Soderlund M , Zhang X *et al*. (2015) Decreased splenic CD4(+) T‐lymphocytes in apolipoprotein M gene deficient mice. Biomed Res Int 2015, 293512. 26543853 10.1155/2015/293512 PMC4620415

[22] Yu Y , Zheng L , Liang Y , Pan L , Zhang J , Wei J , Yu M and Luo G (2015) Establishment of duplex fluorescence RT‐PCR for identification of apolipoprotein M gene knockout mice (In Chinese). Chin J Clin Lab Sci 33, 412–414.

[23] Pan L , Zheng L , Zhang J , Yu Y , Yao S , Yu M , Feng Y and Luo G (2015) Development of a duplex fluorescence RT‐PCR assay for identifying SR‐BI gene knockout mice (In Chinese). Tianjin Med J 43, 732–734.

[24] Zhang X , Goncalves R and Mosser DM (2008) The isolation and characterization of murine macrophages. Curr Protoc Immunol 83, 14.1.1–14.1.14. 10.1002/0471142735.im1401s83 PMC2834554 19016445

[25] Cox GW , Mathieson BJ , Gandino L , Blasi E , Radzioch D and Varesio L (1989) Heterogeneity of hematopoietic cells immortalized by v‐myc/v‐raf recombinant retrovirus infection of bone marrow or fetal liver. J Natl Cancer Inst 81, 1492–1496. 2778838 10.1093/jnci/81.19.1492

[26] Lu J , Allred CC and Jensen MD (2018) Human adipose tissue protein analyses using capillary western blot technology. Nutr Diabetes 8, 26. 29695704 10.1038/s41387-018-0030-4 PMC5916899

[27] Brown MS and Goldstein JL (1983) Lipoprotein metabolism in the macrophage: implications for cholesterol deposition in atherosclerosis. Annu Rev Biochem 52, 223–261. 6311077 10.1146/annurev.bi.52.070183.001255

[28] Lewis GF and Rader DJ (2005) New insights into the regulation of HDL metabolism and reverse cholesterol transport. Circ Res 96, 1221–1232. 15976321 10.1161/01.RES.0000170946.56981.5c

[29] Fielding CJ and Fielding PE (1995) Molecular physiology of reverse cholesterol transport. J Lipid Res 36, 211–228. 7751809

[30] Rader DJ (2007) Mechanisms of disease: HDL metabolism as a target for novel therapies. Nat Clin Pract Cardiovasc Med 4, 102–109. 17245404 10.1038/ncpcardio0768

[31] Portioli Silva EP , Peres CM , Roberto Mendonca J and Curi R (2004) NBD‐cholesterol incorporation by rat macrophages and lymphocytes: a process dependent on the activation state of the cells. Cell Biochem Funct 22, 23–28. 14695650 10.1002/cbf.1048

[32] Song W , Wang W , Wang Y , Dou L , Chen L and Yan X (2012) A New assay of cholesterol efflux rate in human peripheral blood monocyte cells by fluorescent labeled cholesterol (In Chinese). Chin J Arteriosclerosis 20, 749–754.

[33] Yu XH , Fu YC , Zhang DW , Yin K and Tang CK (2013) Foam cells in atherosclerosis. Clin Chim Acta 424, 245–252. 23782937 10.1016/j.cca.2013.06.006

[34] Borup A , Christensen PM , Nielsen LB and Christoffersen C (2015) Apolipoprotein M in lipid metabolism and cardiometabolic diseases. Curr Opin Lipidol 26, 48–55. 25551802 10.1097/MOL.0000000000000142

[35] Cao B , Ye YZ , Rui J , Li MQ , Wang W , Wei LY and Jiao GQ (2013) A single‐nucleotide polymorphism in the proximal promoter region of the apolipoprotein M gene is associated with dyslipidaemia but not increased coronary artery diseases in Chinese populations. Lipids Health Dis 12, 184. 24341666 10.1186/1476-511X-12-184 PMC3903071

[36] Aung LH , Yin RX , Wu DF , Yan TT , Li Q , Wu JZ , Lin WX , Liu CW and Pan SL (2013) Association of the apolipoprotein M gene polymorphisms and serum lipid levels. Mol Biol Rep 40, 1843–1853. 23086303 10.1007/s11033-012-2240-5

[37] Trigatti B , Rigotti A and Krieger M (2000) The role of the high‐density lipoprotein receptor SR‐BI in cholesterol metabolism. Curr Opin Lipidol. 11, 123–131. 10787173 10.1097/00041433-200004000-00004

[38] Rigotti A , Miettinen HE and Krieger M (2003) The role of the high‐density lipoprotein receptor SR‐BI in the lipid metabolism of endocrine and other tissues. Endocr Rev 24, 357–387. 12788804 10.1210/er.2001-0037

[39] Feng YH , Zheng L , Wei J , Yu MM , Zhang J , Luo GH and Xu N (2018) Increased apolipoprotein M induced by lack of scavenger receptor BI is not activated via HDL‐mediated cholesterol uptake in hepatocytes. Lipids Health Dis 17, 200. 30144814 10.1186/s12944-018-0849-7 PMC6109342

[40] Tian K , Xu Y , Sahebkar A and Xu S (2020) CD36 in Atherosclerosis. Pathophysiol Mech Therap Implicat 22, 59. 10.1007/s11883-020-00870-8 32772254

[41] Febbraio M , Podrez EA , Smith JD , Hajjar DP , Hazen SL , Hoff HF , Sharma K and Silverstein RL (2000) Targeted disruption of the class B scavenger receptor CD36 protects against atherosclerotic lesion development in mice. J Clin Invest 105, 1049–1056. 10772649 10.1172/JCI9259 PMC300837

[42] Shi Y , Lam SM , Liu H , Luo G , Zhang J , Yao S , Li J , Zheng L , Xu N , Zhang X *et al*. (2020) Comprehensive lipidomics in apoM(‐/‐) mice reveals an overall state of metabolic distress and attenuated hepatic lipid secretion into the circulation. J Genet Genomics 47, 523–534. 33309167 10.1016/j.jgg.2020.08.003

[43] Kurano M , Tsukamoto K , Hara M , Ohkawa R , Ikeda H and Yatomi Y (2015) LDL receptor and ApoE are involved in the clearance of ApoM‐associated sphingosine 1‐phosphate. J Biol Chem 290, 2477–2488. 25505264 10.1074/jbc.M114.596445 PMC4303696

[44] Frambach SJCM , De Haas R , Smeitink JAM Rongen GA , Russel FGM and Schirris TJJ (2020) Brothers in Arms: ABCA1‐ and ABCG1‐mediated cholesterol efflux as promising targets in cardiovascular disease treatment. Pharmacol Rev 72, 152–190. 31831519 10.1124/pr.119.017897

[45] He P , Gelissen IC and Ammit AJ (2020) Regulation of ATP binding cassette transporter A1 (ABCA1) expression: cholesterol‐dependent and ‐ independent signaling pathways with relevance to inflammatory lung disease. Respir Res 21, 250. 32977800 10.1186/s12931-020-01515-9 PMC7519545

[46] Sparrow CP , Baffic J , Lam MH , Lund EG , Adams AD , Fu X , Hayes N , Jones AB , Macnaul KL , Ondeyka J *et al*. (2002) A potent synthetic LXR agonist is more effective than cholesterol loading at inducing ABCA1 mRNA and stimulating cholesterol efflux. J Biol Chem 277, 10021–10027. 11790770 10.1074/jbc.M108225200

[47] Venkateswaran A , Laffitte BA , Joseph SB , Mak PA , Wilpitz DC , Edwards PA and Tontonoz P (2000) Control of cellular cholesterol efflux by the nuclear oxysterol receptor LXR alpha. Proc Natl Acad Sci USA 97, 12097–12102. 11035776 10.1073/pnas.200367697 PMC17300

[48] Sukhorukov VN and Khotina VA (2020) Lipid metabolism in macrophages: focus on atherosclerosis. Biomedicines 8, 262. 10.3390/biomedicines8080262 PMC7459513 32752275

[49] Guo C , Ma R , Liu X , Chen T , Li Y , Yu Y , Duan J , Zhou X , Li Y and Sun Z (2018) Silica nanoparticles promote oxLDL‐induced macrophage lipid accumulation and apoptosis via endoplasmic reticulum stress signaling. Sci Total Environ 631–632, 570–579. 10.1016/j.scitotenv.2018.02.312 29533793

