# Supplementary material for: Apolipoprotein M promotes cholesterol uptake and efflux from mouse macrophages
Source: FEBS Open Bio. 2021 May 2;11(6):1607–20. doi: 10.1002/2211-5463.13157 (PMC8167864; doi:10.1002/2211-5463.13157)
Supplement: Supplementary file 1 — Fig. S1. Wright staining of peritoneal macrophages from WT mice. Scale bar: 50 μm. Fig. S2. ApoM protein levels in macrophage, Ana‐1, Kidney, and liver detected by western blot based on capillary electrophoresis technology. A pack with 25 capillary cartridges was used in this study. ApoM and β‐actin protein levels were detected by 12–230 kDa pre‐filled plates, respectively. The blots in Fig. 2B in main text are from blots which are framed. Fig. S3. Agarose gel electrophoresis of apoM PCR product derived from Ana‐1 cells, peritoneal macrophages, liver, and kidney from WT mice. A1‐A6: Ana‐1. M1‐M6: Macrophage. L1‐L6: Liver. K1‐K6: Kidney. The blots in green box were used in Fig. 2C. [file FEB4-11-1607-s001.pdf]

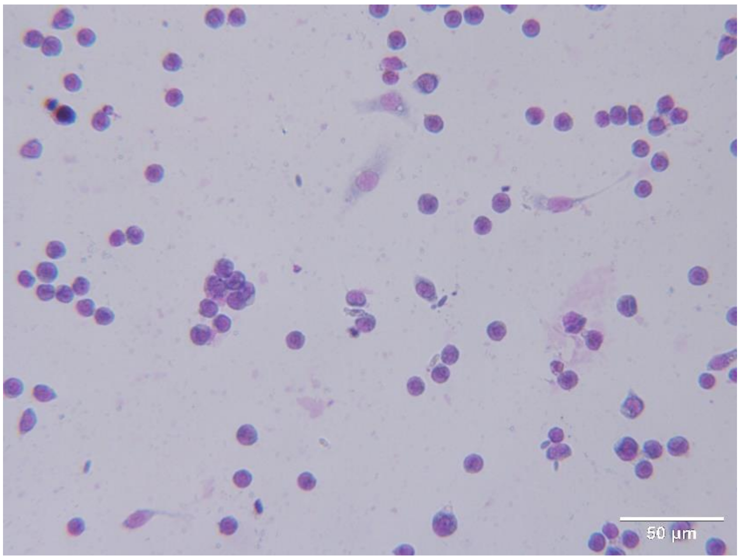

Fig. S1. Wright staining of peritoneal macrophages from WT mice. Scale bar: 50 μm.

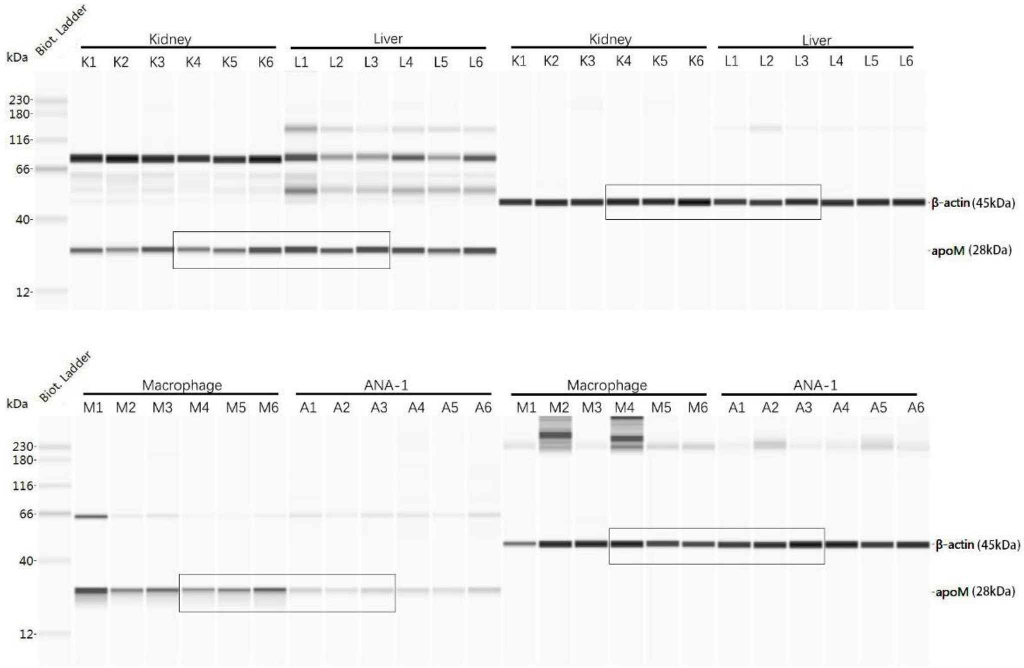

Fig. S2. ApoM protein levels in macrophage, Ana-1, Kidney, and liver detected by western blot based on capillary electrophoresis technology. A pack with 25 capillary cartridges was used in this study. ApoM and β-actin protein levels were detected by 12-230 kDa pre-filled plates, respectively. The blots of in Figure 2B in main text are from blots which are framed.

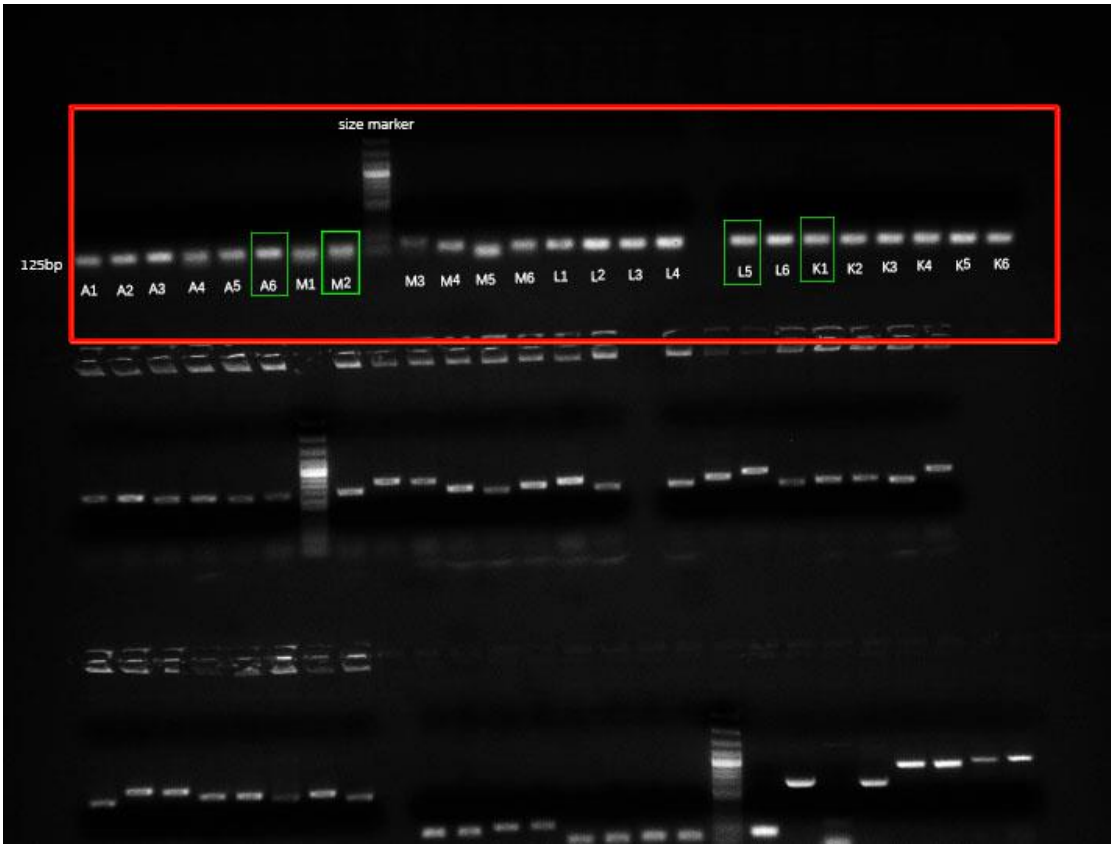

Fig. S3. Agarose gel electrophoresis of apoM PCR product derived from Ana-1 cells, peritoneal macrophages, liver, and kidney from WT mice. A1-A6: Ana-1. M1-M6: Macrophage. L1-L6: Liver. K1-K6: Kidney. The blots in green box were used in Figure 2C.
